# Gender Disparities in Authorship of Invited Manuscripts During the COVID-19 Pandemic

**DOI:** 10.1089/whr.2021.0023

**Published:** 2021-05-25

**Authors:** Cristal Brown, Tessa K. Novick, Elizabeth A. Jacobs

**Affiliations:** ^1^ Department of Medicine, University of Texas Austin Dell Medical School, Austin, Texas, USA.; ^2^ Vice President of Research and Interim Director for Center for Outcomes Research and Evaluation, Maine Medical Center Research Institute, Scarborough, Maine, USA.

**Keywords:** COVID-19, gender disparity, authorship, invited manuscripts

## Abstract

***Introduction:*** Women have historically been under-represented in medical literature, particularly prominent in authorship of invited commentaries. With the instantaneous change in work environment forcing Americans to adapt to working at home, many theorize that women will be more adversely affected due to traditional concepts of women being more responsible for the home in addition to work responsibilities.

***Objective:*** Understand how women contributed to coronavirus disease 2019 (COVID-19) literature early in the pandemic.

***Methods:*** Cross-sectional analysis of manuscripts published in three high-impact U.S. medical journals from February to May 2020 was performed. We used log-binomial regression to examine for an association between COVID-19 status and likelihood of having at least one female first author, and evaluated for effect modification according to whether the manuscript was invited.

***Results:*** Among 980 manuscripts, 313 (31.9%) listed at least one female first author, 203 were written on COVID-19 (20.7%), and 144 (14.7%) were invited. There was no association between COVID-19 status and having at least one female first author overall (adjusted risk ratio [RR] 0.93, 95% confidence interval [CI] 0.72–1.19). The relationship between COVID-19 status and first-author sex was 0.23 (95% CI 0.06–0.92) for invited manuscripts and 1.04 (95% CI 0.81–1.35) for noninvited manuscripts (*p* for interaction 0.02).

***Discussion:*** We demonstrate that women were not less likely to be first authors on COVID-19 manuscripts but were less likely to be first authors on invited COVID-19 manuscripts. Early career female researchers are the most vulnerable for inability to meet metrics for promotion, accounting for the continued under-representation of women in senior academic roles. COVID-19 has the potential to exacerbate this disparity.

## Introduction

Work environments changed dramatically and instantaneously when coronavirus disease 2019 (COVID-19) emerged as a global health threat. Many Americans had to adapt to working at home overnight,^1^ raising concerns about employees' abilities to remain productive in a merged home–work environment. Many theorized that women would be more adversely affected as working women take care of home responsibilities more than men based on traditional concepts of family.^2,3^ This is true for women in academic medicine as well.^4^

Women have historically been under-represented in academic medicine. While there have been recent improvements in equality in assistant professor ranks, women remain under-represented at nearly every other stage.^5,6^ Female under-representation is particularly prominent in authorship, and women are less likely to be authors of invited commentaries. One reason for under-representation is related to women not being as professionally networked or recognized as thought leaders as men.^7–9^

We sought to understand how women contributed to COVID-19 literature early in the pandemic. We hypothesized that COVID-19 manuscripts would be less likely to include at least one female first or last author, and the female under-representation would be greater for invited COVID-19 manuscripts.

## Methods

We conducted a cross-sectional study of a sample of manuscripts published in three high-impact medical journals from February to May 2020: the Journal of the American Medical Association (JAMA), the New England Journal of Medicine (NEJM), and Annals of Internal Medicine (Table 1). We chose this time period as the science surrounding the pandemic rapidly grew due to the global impact of the recently discovered, novel virus and early literature surrounding gender inequality in COVID-19 publication suggested higher percentage of women authors in high-impact journals (impact factor >7) compared with low-impact journals (impact factor <2).^10^ For each manuscript, we collected data on first- and last-author sex, digital object identifier, whether the manuscript was on COVID-19, whether the manuscript was invited, and publication month. We excluded manuscripts with unknown first- or last-author sex (*n* = 32), leaving a sample size of *n* = 980.

**Table 1.** Categorization of Article Types and Invited Status by Journal

Journal	Invited manuscripts	Not-invited manuscripts
JAMA	Editorials	Viewpoints A Piece of My Mind Poetry and Medicine Comment and Response Clinical Update Clinical Challenge The Arts and Medicine Evidence Report Quick Uptake Reviews^a^ Clinical Guideline Synopsis^a^ Diagnostic Test Interpretation^a^
NEJM	Editorials Reviews Clinical Implications of Basic Science Clinical Practice Clinical Decisions	Original Research Medicine and Society Correspondence Special report Clinical Problem-Solving Perspectives^a^ Sounding Board^a^ Health Policy Report^a^
Annals	Editorials	Beyond the Guidelines Opinion On Being a Doctor *Ad Libitum* In the Clinic Position Papers Ideas and opinions Understanding Clinical Research Letters: Comments Research and Reporting Methods Special Articles Original Articles

^a^ These article types are sometimes solicited or require editor permission for submission. In the primary analysis, they were defined as noninvited. In sensitivity analysis, they were defined as invited.

Annals, Annals of Internal Medicine; JAMA, Journal of the American Medical Association; NEJM, New England Journal of Medicine.

Each study author abstracted data from one journal. First- and last-author sex was ascertained by looking up each author on the internet. Author sex was determined in sequential order of

(1) explicit statement of male or female sex,
(2) use of gender-specific pronouns,
(3) physical appearance, and
(4) email corresponding author.

First-author sex was defined as having at least one female first author (yes/no). Last-author sex was defined as having at least one female last author (yes/no). These designations were made based on numerous articles with equal contributions of multiple first and last authors. Articles were defined as COVID or non-COVID according to whether they covered topics related to the COVID-19 pandemic. Invited status was defined according to author guidelines on each journal's website, and after confirming with the editorial board for each journal. If a journal indicated that an article type was sometimes invited, but not always, it was defined as not invited. Each study author's work was verified by a second study author, who reviewed a random sample of the manuscripts to confirm the information as recorded correctly. If an error was identified on the random sample, all collected data were reviewed to confirm accuracy.

We compared characteristics of manuscripts between those with and without at least one female first author using chi-square tests for categorical variables. We used log-binomial regression to examine the association between COVID-19 status and likelihood of having at least one female first author, and adjusted for invited status, journal, and publication month. Among manuscripts with more than one author (*n* = 704), we examined the association between COVID-19 status and likelihood of having at least one female last author, and adjusted for invited status, journal, and publication month. We evaluated for potential effect modification by invited status by including an interaction term for COVID-19 × invited. Among manuscripts with more than one author, we used log-binomial regression to examine associations between last-author sex and first-author sex, and adjusted for COVID-19 status, invited status, journal, and publication month. As a sensitivity analysis, we classified article types that were sometimes invited as invited instead of not invited, and repeated the primary analysis. We used Poisson's regression with robust estimation of variance for any models that failed to converge. All analyses were performed using Stata version 14.2 (StataCorp, College Station, TX). A *p*-value <0.05 was considered statistically significant.

## Results

Among 980 manuscripts, 313 (31.9%) listed at least one female first author, 203 (20.7%) were written on COVID-19, and 144 (14.7%) were invited (Table 2). Among 704 manuscripts with more than one author, 197 (28.0%) listed a female last author. Compared with manuscripts without female first authors, those with at least one female first author were less likely to be published in NEJM (41.9% vs. 50.2%, *p* = 0.04). When comparing at least one female first author for the total number of manuscripts contributed by each journal, JAMA had 136/375 (36.3%), Annals had 46/139 (33.1%), and NEJM had 131/466 (28.1%).

**Table 2.** Manuscript Characteristics According to the Presence of at Least One Female First Author in All COVID-19 Manuscripts

Characteristic	Total (N)	No female first authors n = 667	≥1 Female first authors n = 313	*p*
COVID, *n* (%)	203	142 (21.3%)	61 (19.5%)	0.52
Invited, *n* (%)	144	103 (15.5%)	41 (13.1%)	0.33
Issue month				0.34
February	207	134 (20.1%)	73 (23.3%)	
March	212	148 (22.2%)	64 (20.4%)	
April	254	182 (27.3%)	72 (23.0%)	
May	307	203 (30.4%)	104 (33.2%)	
Journal				0.04
JAMA	375	239 (35.8%)	136 (43.5%)	
NEJM	466	335 (50.2%)	131 (41.9%)	
Annals	139	93 (13.9%)	46 (14.7%)	

COVID-19, coronavirus disease 2019.

There were 41 (28.5%) female first authors among 144 invited manuscripts, and 272 (32.5%) female first authors among 836 noninvited manuscripts. There was no association between COVID-19 status and having at least one female first author overall (unadjusted risk ratio [RR] 0.93, 95% confidence interval [CI] 0.73–1.17; adjusted RR 0.93, 95% CI 0.72–1.19). However, the adjusted RR for the relationship between COVID-19 status and first-author sex was 0.23 (95% CI 0.06–0.92) for invited manuscripts and 1.04 (95% CI 0.81–1.35) for noninvited manuscripts (*p* for interaction 0.02; Table 3).

**Table 3.** Likelihood of Having at Least One Female First Author and at Least One Female Last Author Comparing COVID-19 with Non-COVID-19 Manuscripts Stratified by Invited Status

	Overall		Invited manuscripts		Noninvited manuscripts	
	*N*	RR (95% CI)	*n*	RR (95% CI)	*n*	RR (95% CI)
First author	980	0.93 (0.72–1.19)	144	0.23 (0.06–0.92)	836	1.04 (0.81–1.35)
Last author	704	0.88 (0.65–1.20)	93	0.38 (0.12–1.16)	611	1.01 (0.74–1.39)

*p*-Value for interaction for female first authors = 0.02; *p* for interaction for female last authors 0.07. The overall model was adjusted for invited status, journal, and publication month. The stratified models were adjusted for journal and publication month.

CI, confidence interval; RR, risk ratio.

Among manuscripts with more than one author, there was no association between COVID-19 status and having at least one female last author (adjusted RR 0.88, 95% CI 0.65–1.20). The adjusted RR for the relationship between COVID-19 status and last-author sex was 0.38 (95% CI 0.12–1.16) for invited manuscripts and 1.01 (95% CI 0.74–1.39) for noninvited manuscripts (*p* for interaction 0.07; Table 3). There was no association between last-author sex and first-author sex (adjusted RR 1.2, 95% CI 0.99–1.56).

In a sensitivity analysis that classified sometimes invited manuscripts as invited, findings for the association between COVID-19 status and first-author sex were similar to the primary analysis (adjusted RR 0.90, 95% CI 0.70–1.15).

## Discussion

The results of this cross-sectional study are consistent with recent literature of female authorship in early COVID-19 supporting gender inequality in medical publication.^10–12^ As seen in both COVID and non-COVID publications, female first authors are represented in ∼30% of manuscripts.^7,10^ We demonstrate that women were not less likely to be first authors on COVID-19 manuscripts overall, but were less likely to be first authors on invited COVID-19 manuscripts.

Our study found that there was no difference in the overall contribution of women to the COVID-19 literature, similar to results observed by Andersen et al.^11^ but contrary to reports of fewer preprints and initiated projects suggested by other early assessments of gender inequality in the COVID-19 literature.^12^ Our finding suggests that women are findings ways to remain productive in research despite this unexpected and sustained convergence of home–work environment. Women appear to be finding a balance of home and work responsibilities that are not adversely affecting productivity as initially suggested.

Yet, our study is consistent with female first authors being more adversely affected and past findings that women are less likely to be first authors on invited commentaries.^13,14^ In a case–control study of invited commentaries that included 2459 medical journals, compared with male counterparts of similar expertise, seniority, and publication metrics, women were 21% less likely to author an invited commentary. The disparity increased as the female authors' seniority increased, by an additional 3% for every additional 10 years of active publication.^15^ This disparity is significant, since being invited to write a commentary or editorial is associated with being promoted as a thought leader in a field, and often results in additional opportunities that facilitate academic promotion.

Of note, having at least one female last author was not statistically significant for COVID-19 invited manuscripts. This finding occurs despite women being less likely to participate as first authors in the same manuscript type. The last author position is more likely to be a senior author and the individual invited to provide a commentary as a thought leader. Irrespective of last-author sex, they are less likely to invite female colleagues compared with male to participate in these invited commentaries.

Disparities in authorship gender are potentially related to two factors. First, those who are inviting authors of commentaries: editors, editorial boards, and reviewing editors; are disproportionately male, and men are more likely to be networked with other men.^16,17^ This editorial male predominance may result in decreased invitations for women in commentaries, creating a preferential male voice. Second, women are not recognized as experts as often as men.^18,19^ We are more likely to act on unconscious biases when we are under stress, particularly time pressure.^20^ The COVID-19 pandemic has placed incredible stress on all of us, including editors of medical and scientific journals managing an onslaught of COVID-19-related submissions and rapidly needing to consider who are experts to speak to the pandemic and provide commentary on the evolution of the science during this tumultuous time. Women declining invitations for COVID-19 commentary due to altered work environment and responsibilities may be another contributing factor to our findings.

Early career female researchers are the most vulnerable to challenges in meeting the metrics they need for promotion. Unless we support them, the under-representation of women in senior academic roles will persist. COVID-19 has the potential to exacerbate this disparity by the tremendous economic burden that has been created.^3^ In academic medicine, there has already been a reduction in funding for research. With limited resources, early career female clinician-researchers are at risk of having difficulties obtaining funding to continue their career development and progress in academic medicine. To combat the possibility of growing under-representation for the transition of early career female researchers to promotion as senior researchers, it is important that early career female authors have equal opportunities for authorship in both invited and noninvited manuscripts.

Some limitations bear mention. First, our ascertainment of sex was limited to looking up each author on the internet. This method was dependent on the authors' internet presence because this information is not currently collected by journals or publicly available. We acknowledge that sex at birth and gender are separate constructs, and we were not able to ascertain self-identified gender, or include an option for those who identify as nonbinary. Additional research is needed with self-reported sex and gender to further explore the gender disparities found in this study. Second, while we focused on the early effects of the COVID-19 pandemic, as this crisis persists, the long-term effects on gender disparity in authorship are unclear as research is ongoing and takes considerable time to reach publication for further assessment. Finally, our study was cross-sectional and observational, limiting our ability to establish a causal relationship or temporality.

## Conclusion

In conclusion, our study suggests that women were equally likely to contribute to the research literature on COVID-19 early in the pandemic, but were less likely to be first authors in invited COVID-19 manuscripts. Inequities in opportunity for female authors, especially during times of crisis, could have long-term career implications. The manifest questions are why authorship disparities persist, and how to intervene. Data collection on self-reported author characteristics and additional research into the mechanisms underlying these disparities are needed.

## Abbreviations Used

JAMA: Journal of the American Medical Association
CI: confidence interval
COVID-19: coronavirus disease 2019
NEJM: New England Journal of Medicine
RR: risk ratio
